# The Resource Identification Initiative: A cultural shift in publishing

**DOI:** 10.1002/cne.23913

**Published:** 2015-11-19

**Authors:** Anita Bandrowski, Matthew Brush, Jeffery S. Grethe, Melissa A Haendel, David N. Kennedy, Sean Hill, Patrick R. Hof, Maryann E. Martone, Maaike Pols, Serena C. Tan, Nicole Washington, Elena Zudilova‐Seinstra, Nicole Vasilevsky

**Affiliations:** ^1^ Center for Research in Biological SystemsUCSDLa JollaCaliforniaUSA; ^2^ Oregon Health & Science University (OHSU) LibraryDepartment of Medical Informatics & Clinical EpidemiologyPortlandOregonUSA; ^3^ University of Massachusetts Medical School, Department of PsychiatryUniversity of Massachusetts Medical SchoolWorcesterMassachusettsUSA; ^4^ Karolinska InstitutetStockholmSweden; ^5^ Fishberg Department of Neuroscience and Friedman Brain InstituteIcahn School of Medicine at Mount SinaiNew YorkNew YorkUSA; ^6^ Scientific Outreach ExecutiveLondonUK; ^7^ John Wiley & SonsHobokenNew JerseyUSA; ^8^ Lawrence Berkeley National LaboratoryBerkeleyCaliforniaUSA; ^9^ ElsevierAmsterdamthe Netherlands

**Keywords:** research resources, Resource Identification Initiative, identifiability

## Abstract

A central tenet in support of research reproducibility is the ability to uniquely identify research resources, i.e., reagents, tools, and materials that are used to perform experiments. However, current reporting practices for research resources are insufficient to identify the exact resources that are reported or to answer basic questions such as “How did other studies use resource X?” To address this issue, the Resource Identification Initiative was launched as a pilot project to improve the reporting standards for research resources in the Methods sections of articles and thereby improve identifiability and scientific reproducibility. The pilot engaged over 25 biomedical journal editors from most major publishers, as well as scientists and funding officials. Authors were asked to include Research Resource Identifiers (RRIDs) in their articles prior to publication for three resource types: antibodies, model organisms, and tools (i.e., software and databases). RRIDs are assigned by an authoritative database, for example, a model organism database for each type of resource. To make it easier for authors to obtain RRIDs, resources were aggregated from the appropriate databases and their RRIDs made available in a central Web portal (http://scicrunch.org/resources). RRIDs meet three key criteria: they are machine‐readable, free to generate and access, and are consistent across publishers and journals. The pilot was launched in February of 2014 and over 300 articles have appeared that report RRIDs. The number of journals participating has expanded from the original 25 to more than 40, with RRIDs appearing in 62 different journals to date. Here we present an overview of the pilot project and its outcomes to date. We show that authors are able to identify resources and are supportive of the goals of the project. Identifiability of the resources post‐pilot showed a dramatic improvement for all three resource types, suggesting that the project has had a significant impact on identifiability of research resources. J. Comp. Neurol. 524:8–22, 2016. © 2015 The Authors The Journal of Comparative Neurology Published by Wiley Periodicals, Inc.

Research resources, defined here as the reagents, materials, and tools used to produce the findings of a study, are the cornerstone of biomedical research. However, as has long been bemoaned by database curators and investigated by Vasilevsky et al. (2013), it is difficult to uniquely identify these resources in the scientific literature. This study found that researchers did not include sufficient detail for unique identification of several key research resources, including model organisms, cell lines, plasmids, knockdown reagents, or antibodies. In most cases, authors provided insufficient metadata about the resource to conclusively identify the particular resource, e.g., a nonunique set of attributes with no catalog or stock number. It should be noted that the authors were, generally speaking, following the reporting guidelines offered by the journals. Such guidelines traditionally state that authors should include the company name and city in which it was located for the resources used in the study. Further, even when uniquely identifying information was provided (e.g., a catalog number for a particular antibody), the vendor may have gone out of business, the particular product may no longer be available, or its catalog information may have changed. Given that in these cases a human cannot find which resources were used, an automated agent, such as a search engine or text mining tools, will also not be able to identify the resources.

Because current practices for reporting research resources within the literature are inadequate, nonstandardized, and not optimized for machine‐readable access, it is currently very difficult to answer very basic questions about published studies such as “What studies used the transgenic mouse I am interested in?” These types of questions are of interest to the biomedical community, which relies on the published literature to identify appropriate reagents, troubleshoot experiments, and aggregate information about a particular organism or reagent to form hypotheses about mechanism and function. Such information is also critical to funding agencies that funded a research group to generate a particular tool or reagent; and the resource providers, both commercial and academic, who would like to be able to track the use of these resources in the literature. Beyond this basic utility, identification of the particular research resource used is an important component of scientific reproducibility or lack thereof.

The Resource Identification Initiative (RII) is laying the foundation of a system for reporting research resources in the biomedical literature that will support unique identification of research resources used within a particular study. The initiative is jointly led by the Neuroscience Information Framework (NIF; http://neuinfo.org) and the Oregon Health & Science University (OHSU) Library, data integration efforts occurring as part of the Monarch Initiative (www.monarchinitiative.org), and with numerous community members through FORCE11, the Future of Research Communications and e‐Scholarship, which is a grassroots organization dedicated to transforming scholarly communication through technology. Since 2006, the NIF has worked to identify research resources of relevance to neuroscience. The OHSU group has long‐standing ties to the model organism community, which maintains databases populated by curating the literature and contacting authors to add links between model organisms, reagents, and other data. In a 2011 workshop (see https://www.force11.org/node/4145) held under the auspices of the Linking Animal Models to Human Diseases (LAMHDI) consortium, various stakeholders from this community drafted recommendations for better reporting standards for animal models, genes, and key reagents.

The RII initiative was launched as a result of two planning meetings building off of the recommendations of the LAMHDI workshop. The first was held in 2012 at the Society for Neuroscience meeting with over 40 participants comprising editors, publishers, and funders (sponsored by INCF; http://incf.org). This meeting outlined the problem of incomplete identification of research resources within articles, and the need for a computational solution for identifying and tracking them in the literature. Recognizing that any solution needed to work for both humans and machines, three broad requirements were identified: 1) the standard should be machine‐processable, that is, designed for search algorithms, in addition to human understanding; 2) the information should be available outside the pay wall, so that search algorithms and humans have free access to the information across the biomedical literature; and 3) the standard should be uniform across publishers, to make uptake and usage easier for both human and machines.

A follow‐up workshop at the NIH (https://www.force11.org/node/4857) was held in June of 2013 to gain agreement from this stakeholder group for the design of a pilot that would explore solutions for this problem. A working group, the Resource Identification Initiative, was established through FORCE11 comprised of publishers, journal editors, antibody manufacturers and distributors, biocurators, software tool developers, and foundations. Based on agreements garnered at the June 2013 meeting, the RII designed a pilot project to test implementation of a system for authors submitting manuscripts to identify research resources through the use of a unique identifier, termed a Research Resource Identifier (RRID).

## PILOT PROJECT OVERVIEW

The pilot project limited its focus to three types of resources: primary antibodies, noncommercial software tools/databases, and model organisms. These were chosen because they are a major source of variation across experiments and are used broadly across biomedical research communities. For the purposes of this pilot, a critical aspect was that a relatively complete and authoritative central registry existed that could issue an accession number, as GenBank does for gene sequences. To gain broad agreement among publishers and editors who were concerned about the potential burden on authors and staff, it was agreed that participation in the pilot project would be voluntary for authors, with participation not representing a condition of acceptance for publication. The pilot project was also designed to have minimal requirements for publishers such that modification of manuscript submission systems was not required.

The pilot project was originally designed to run for 6 months, with each of the participating journals agreeing to participate for at least 3 months. The goal was to ensure a large enough sample to understand author behavior: could they and would they do the task, and to obtain sufficiently large participation to demonstrate the utility of RRIDs. Over the minimum 3‐month window, each partner journal would request authors to supply RRIDs in a standard format as a citation to indicate the use of antibodies, software and databases, and model organisms. To be as unambiguous as possible, authors were to include the RRIDs for resources that were utilized in the study and described in the text of the Materials and Methods, but not in the Introduction or Discussion sections, where they might be mentioned in passing but not used in the study. The RRID syntax comprises an accession number assigned by the authoritative database with the prefix “RRID:” prepended (e.g., RRID:AB_2298772 for an antibody). We also requested that non‐open‐source journals include RRIDs in the keyword field, as this field is available for indexing in PubMed outside of pay‐walls. The journals were given flexibility for when and how they wanted to ask authors for these identifiers, namely, at time of submission, during review, or after acceptance. They were not required to modify their instructions to authors or their submission systems. The RII team would be responsible for preparing appropriate materials for requesting RRIDs and for establishing a central portal where these identifiers could be obtained. The RII team also agreed to establish a help desk to assist the authors if they encountered any difficulties.

The pilot project was designed to address four key questions. A set of evaluation criteria was designed for each question:

1. *Participation*: Would authors be willing to add resource identifiers to their publications and register new resources in the system? Participation was evaluated by examining the number of submissions to the participating journals, the rate of author participation in providing RRIDs, the number of new resources registered, and direct feedback from authors.
2. *Performance*: Could authors add these identifiers correctly or would additional editorial or staff oversight be necessary? Performance was measured by a quantitative analysis of RRID correctness by RII curators.
3. *Identifiability*: Would the use of RRIDs improve our ability to identify resources in the literature? Identifiability was measured both pre‐ and post‐pilot in the journals that participated.
4. *Utility*: Will RRIDs be useful to the scientific community? Can the RRIDs as constructed be used to identify all studies that use a particular research resource? To encourage the development of applications, the dataset is being made freely available so that third parties can develop tools to work with RRIDs.

The pilot began in February 2014, with over 25 journals participating. Journals that sent a letter to authors at some stage of the review process included: *Journal of Neuroscience, Brain and Behavior, Journal of Comparative Neurology, Brain Research, Experimental Neurology, F1000Research, PeerJ, Journal of Neuroscience Methods, Neurobiology of Disease*, and the Frontiers group of journals. One journal, *Neuroinformatics*, chose to add the RRIDs to all manuscripts before asking authors to do this. Journals in the Elsevier and BMC groups were participants based on updates to their instructions to authors. Because of the success of the project, it was subsequently extended and is still active as of this writing. The number of journals participating has expanded, and now includes *PLoS Biology* and *PLoS Genetics* as well as multiple immunology journals in the Elsevier family. A list of the participating journals is available on the Force11 Website (https://www.force11.org/RII/SignUp).

One of the primary requirements of the pilot project was to make it as easy as possible for authors to obtain the appropriate identifiers and insert them correctly into their manuscripts. As noted above, the three research resources were chosen because each was covered by an authoritative database (Table 1) that assigned unique IDs and a standard set of metadata to each. However, as can be seen by the length of the list in Table 1, authors could potentially be required to visit several databases to obtain the appropriate identifiers.

**Table 1:** Source Databases and Registries Included in the RII Portal

Resource name	Resource content	Database identifier
ZIRC, Zebrafish Resource Center	Zebrafish stocks	RRID:nif‐0000‐00242
ZFIN, Zebrafish Information Network	Zebrafish nomenclature	RRID:nif‐0000‐21427
RGD, Rat Genome Database	Rat	RRID:nif‐0000‐00134
CGC, Caenorhabditis Genetics Center	Worm stocks	RRID:nif‐0000‐00240
WormBase	Worm nomenclature	RRID:nif‐0000‐00053
IMSR, International Mouse Strain Resource Center	Mouse stocks	RRID:nif‐0000‐09876
BDSC, Bloomington Drosophila Stock Center	Fly stocks	RRID:nif‐0000‐00241
MGI, Mouse Genome Informatics	Mouse nomenclature	RRID:nif‐0000‐00096
BCBC, Beta Cell Biology Consortium	Mouse stocks	RRID:nlx_144143
antibodyregistry.org, Antibody Registry	Antibodies	RRID:nif‐0000‐07730
SciCrunch Registry	Software tools and databases	RRID:nlx_144509

Each database has a weekly or monthly scheduled frequency of update and all new data are released weekly. If available, data from both model organism authorities is served, as well as the list of strains available via particular stock centers. In most cases the stock centers maintain a link between the genotype and the stock center animal identifier.

**Figure 1:**
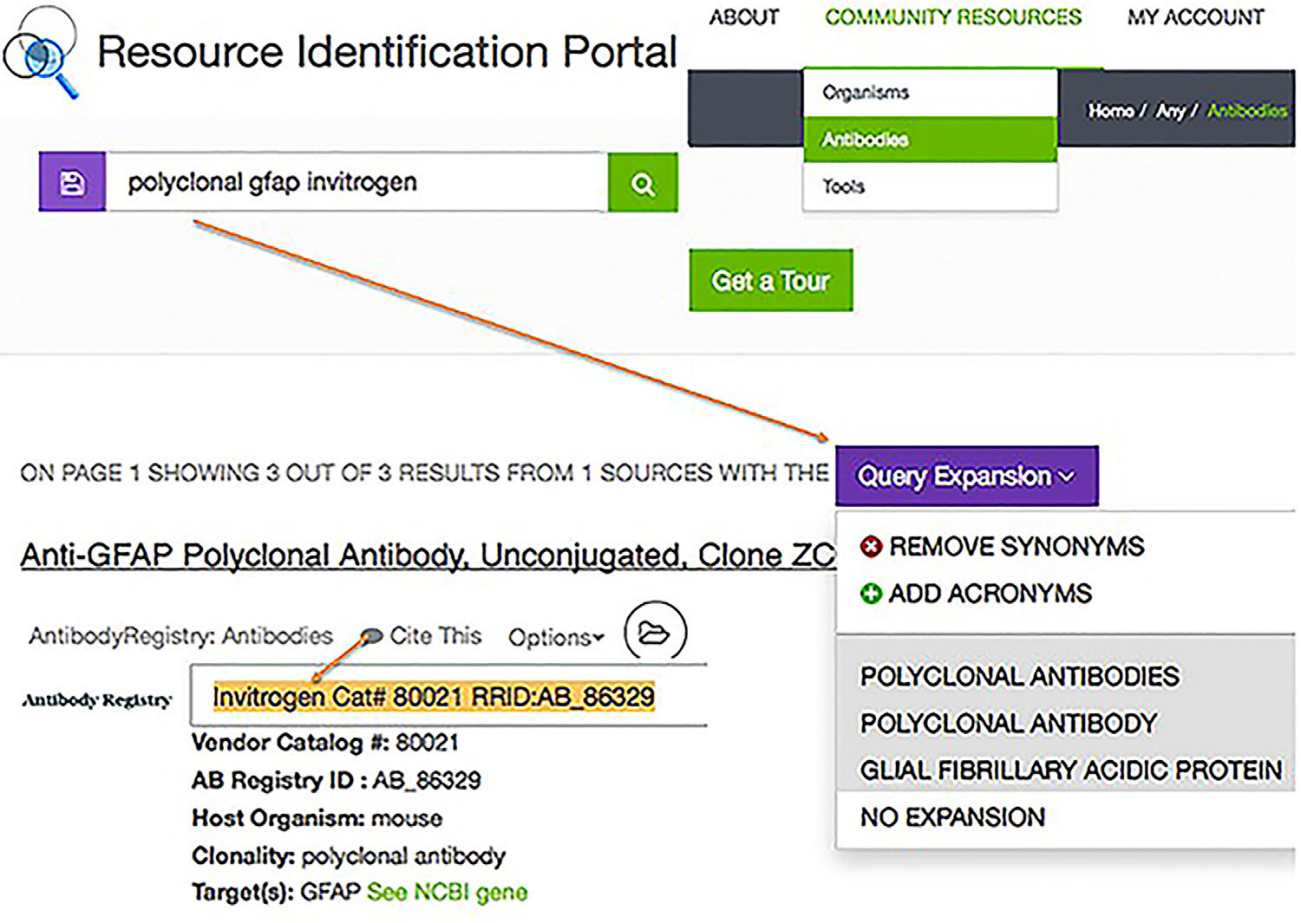
The Resource Identification Initiative portal containing citable Research Resource Identifiers (RRIDs). The workflow for authors is to visit http://scicrunch.org/resources, then select their resource type (see community resources box), type in search terms (note that the system attempts to expand known synonyms to improve search results), and open the “Cite This” dialog box. The dialog shown here displays the Invitrogen catalog number 80021 antibody with the RRID:AB_86329.

To simplify this process, we established a Resource Identification Portal based on the SciCrunch platform, which leverages data aggregation performed by the DISCO aggregation engine (Marenco et al., 2014; http://scicrunch.org/resources; Fig. 1). The portal provides a unified query across different resource databases and displayed the results in a common format. The portal allows search on various facets such as resource name, catalog number, etc. There is a “cite this” link that provides the citation, as it should be reported in the article. The citation generally includes not just the RRID, but a set of appropriate metadata that would identify the vendor and catalog number as well, for example: A polyclonal antibody against tyrosine hydroxylase (TH) (Chemicon, Cat. AB1542, RRID:AB_90755).

## METHODS

SciCrunch was built based on the extensible Neuroscience Information Framework platform described previously (Gardner et al., 2008; Marenco et al., 2014; RRID:nif‐0000‐25673), and the portal infrastructure for RII was developed under an award from NIDDK to create a dkNET portal (RRID:nlx_153866), while the customization of the portal was done by Monarch staff. The data are aggregated from the SciCrunch tool registry, the antibody registry, as well as the model organism community databases and stock centers (Table 1). The data infrastructure allows curators to keep indexes synchronized with the source databases by using an automated crawling engine and new data are released on a weekly basis. All open data from each of these databases is available to download from the source sites, where update frequencies are listed.

The journal editors were provided with recommended instructions to authors (the instructions to authors are available here: https://www.force11.org/node/4856). For antibodies, we only required authors to identify primary antibodies and not secondary or tertiary complexes. For software tools and databases we focused on freely available and generally publicly funded noncommercial tools. For model organisms, we focused on the five commonly used organisms: mouse, rat, zebrafish, fruit fly, and worm. Authors were asked to insert the correct citation for the resource into the text of the Materials and Methods section and in the keywords. A help desk was established by the RII working group that provided help if an author encountered difficulty. In most cases, requests were handled in less than 24 hours.

If a resource was not found via the portal, authors were given the option to submit the resource to obtain an identifier. For antibodies and software/databases, which are found in databases maintained within the NIF, submission was handled through the Resource Identification Portal. For model organisms, the author was referred to the authoritative model organism database for their organism (RGD, MGI, ZFIN, Wormbase, or Flybase). All new submissions were curated by their respective databases and the data were pulled back into the RII portal weekly so that authors could see their newly registered resources in approximately a week.

To evaluate the aims of the pilot project, we tracked the use of RRIDs in published articles and journals. We performed an in‐depth analysis of the first 100 articles found through Google Scholar that reported RRIDs. For each article, we examined the Methods section to determine the correct usage (i.e., if the RRID pointed to the correct resource), the syntactic correctness (i.e., if the author reported the RRID using the correct syntax), and the identifiability of the three resource types. The total number of research resources reported in the first 100 articles reporting RRIDs was determined by manual inspection of each article by two independent curators. A Google Scholar alert was used to track all new articles that contained an RRID, using the search “RRID:”. Each of the first 100 articles was downloaded and examined for the snippets of text surrounding research resources (in the Methods or Data Use sections).

### Curation workflow to determine correct usage of reported RRIDs

To determine if the RRIDs were reported correctly for the three resource types, the following criteria were applied.

- A resource was considered correct if resource reported an RRID and that RRID pointed to the correct resource in the RII portal. This determination was made both by manual search of the RII portal and via the SciCrunch resolving service for each reported RRID (for example, https://scicrunch.org/resolver/RRID:AB_262044).
- A resource was considered incorrect if the reported RRID pointed to a different or nonexising resource in the RII portal or SciCrunch resolving service.
- A resource was considered to have the correct syntax if the resource reference contained an RRID, and the RRID was formatted correctly, had no missing characters or other typos.

### Curation workflow for identifiability of the three resource types

To determine if the three resource types were identifiable in the journal articles that reported RRIDs (post‐pilot), and in articles from the same journals before the pilot started. To select the pre‐pilot articles, articles were selected by performing a PubMed search filtered for each journal and using the first five publications returned that contained the relevant resource types from approximately January–March 2013. The following criteria were applied: Resources (primary antibodies, organisms, and noncommercial tools) were considered identifiable if they contained an accurate RRID or by using the same specific resource identification criteria as described in Vasilevsky et al. (2013). Noncommercial software and databases that were not previously analyzed were considered identifiable if they contained the correct RRID or reported the manufacturer and version number for that tool. Note that we distinguished commercially produced for‐profit software from public or individually produced software (noncommercial).

### Statistical analysis for identifiability of the three resources

Since the data were binomial in that each resource was either identifiable or not, we used a binomial confidence interval strategy for calculating upper and lower 95% confidence intervals (CI) (http://www.danielsoper.com/statcalc3/calc.aspx?id=85, RRID:SCR_013827). Error bars for the corresponding 95% CI are displayed on the graphs. Statistical significance was determined by calculating the z‐score.

## RESULTS

**Figure 2:**
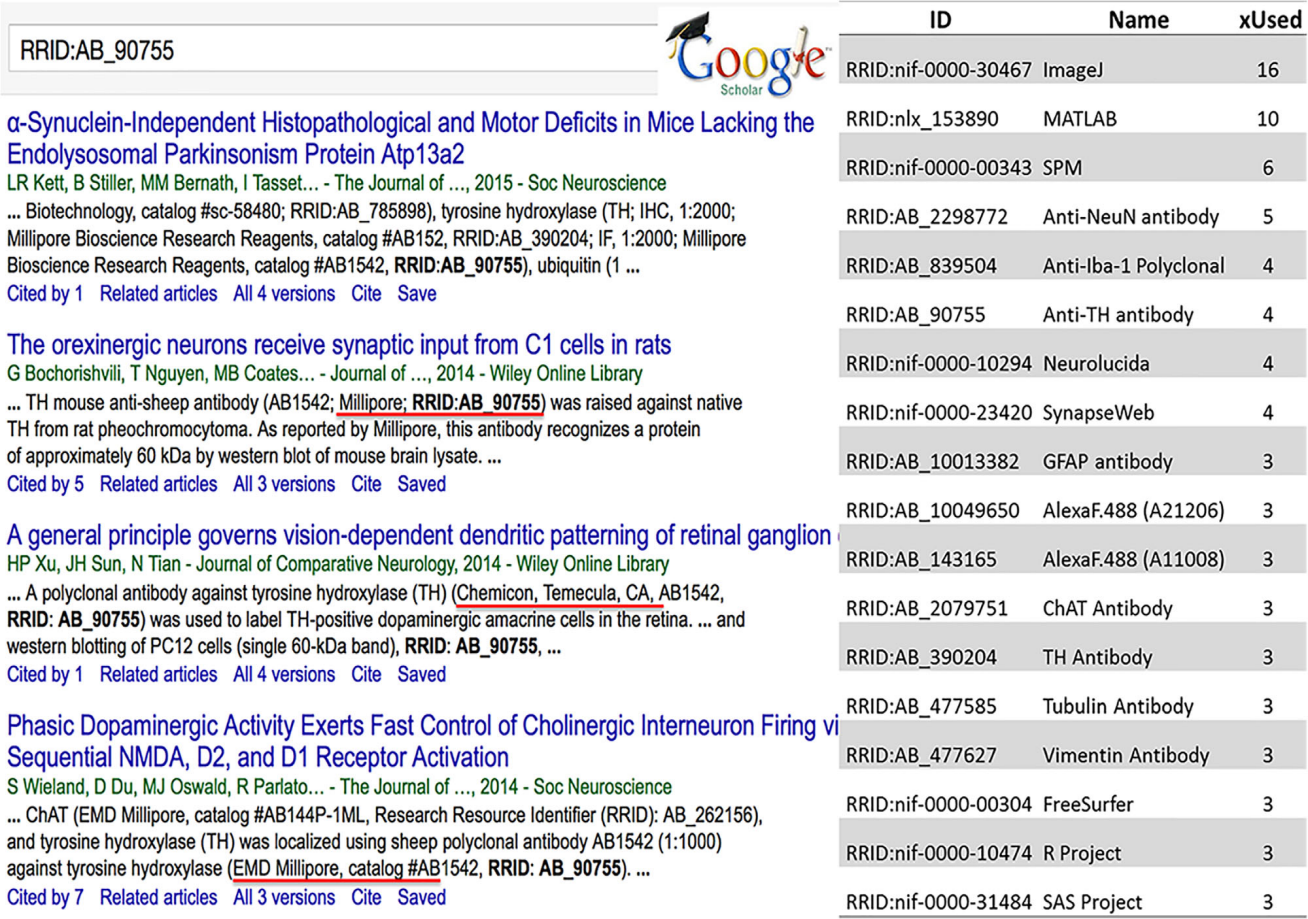
RRIDs found in the published literature. Google Scholar result for the anti‐tyrosine hydroxylase antibody RRID (9/2014; http://scholar.google.com/scholar?q=RRID:AB_90755) and the most frequently reported RRIDs in the first 100 articles, by number of articles using the identifier. All data are available in the Supplementary Table and all identifiers can be accessed in Google Scholar (see also Supplemental Table).

The first RRIDs began appearing in the literature in April of 2014. Although the first article was identified through PubMed, the majority of articles were found via Google Scholar by searching for “RRID.” Google Scholar, unlike PubMed, appears to search the full text of articles, as it returns snippets of text from the materials and methods containing the RRIDs (for example, Fig. 2). A search in PubMed returns very few articles, indicating that most journals were not including the RRIDs outside of the pay‐wall. As these articles start to appear in PubMed Central, where full text search is possible, we anticipate that more articles utilizing RRIDs will be identifiable through the National Library of Medicine. Google Scholar possesses the advantage in that it obtains articles without an embargo period and makes them available for search immediately at the time of publication. In this article, we therefore present analysis based on Google Scholar.

Search via Google Scholar reveals that the RRID prefix is not a unique string, but is an acronym for several entities, most commonly the Renal Risk in Derby clinical study (for example, McIntyre et al., 2012). To return examples of RRIDs requires the use of additional filters, e.g., restricting search to the years 2014 and later. The combination of the RRID prefix with the resource accession number is unique, however, in that searching for a particular RRID, for example RRID:AB_90755, returns only articles that use this research resource (Fig. 2).

The first 100 articles were published in 16 journals and included 562 RRIDs reported by authors. The bulk of the identifiers (490) came from two journals, the *Journal of Comparative Neurology* (JCN) and the *Journal of Neuroscience*, as these two journals were first to participate, both starting the pilot in early February of 2014.

### Outcome #1: Participation

As of March 1, 2015 there were 312 articles published with at least one RRID, from 44 unique journals (Supplementary Table 1 shows the updated list of journals and a count for each), indicating that hundreds of authors have participated in the pilot project even though it is voluntary. Table 2 shows the different mechanisms and timing of contact for authors by different journals. Informal feedback from the editors and authors via the help requests and other correspondence indicates that authors who are attempting to find RRIDs are supportive of the aims of the project and readily able to find the correct RRIDs.

**Table 2:** Journal Practices in Contacting Authors

Journal	Submission	Review	Acceptance	Compliance	Notes
Journal of Neuroscience	Letter (1175)	Letter (163)	Letter (26)	∼12%	Asking at different stages has no effect on rate of compliance
Journal of Comparative Neurology	Working with Author	Working with Author	Working with Author	>90%	Published an editorial and has a history of proper antibody identification back to 2006
Brain and Behavior			Letter (∼100)	∼25%	Letters started to be sent out in April 2014, at times the editor followed up with authors, did not keep exact records
Neuroinformatics			Staff looks up data	100%	Journal has a section for tools used in the study, which now includes RRIDs, several papers incorporated RRIDs prior to staff intervention
F1000 Research			Letter (∼50)	12%	Approximate figure from editor
Brain Research		Letter (671)		1%	Authors receive automatically generated letters with multiple instructions, including RII guidelines. Authors are asked to incorporate RRIDs or database identifiers (overall compliance 1%; for RRIDs < 1%).
Journal of Neuroscience Methods		Letter (314)		1%	Authors receive automatically generated letters with multiple instructions, including RII guidelines. Authors are asked to incorporate RRIDs or database identifiers (overall compliance 1%; for RRIDs < 1%).
Neurobiology of Disease		Letter (291)		3%	Authors receive automatically generated letters with multiple instructions, including RII guidelines. Authors are asked to incorporate RRIDs or database identifiers (overall compliance 3%; for RRIDs 2%).
Experimental Neurology		Letter (297)		3%	Authors receive automatically generated letters with multiple instructions, including RII guidelines. Authors are asked to incorporate RRIDs or database identifiers (overall compliance 3%; for RRIDs < 1%).

Different journals chose to contact authors at different stages of the publishing cycle and assist in the addition of RRIDs via different mechanisms. The participation rate was by far the lowest with only instructions to authors; these journals are not included in this table (for example BMC) and had < 1% participation rates. When authors were asked by a blanket mailing containing instructions, participation rates ranged between 1 and 15%. Participation was very high if the editorial staff asked authors directly or suggested identifiers for their manuscript. Note that in some cases only an approximation could be made by the participating journals.

One journal, the *Journal of Neuroscience*, sent authors letters asking for their participation during different periods of the publication cycle. There did not appear to be a more advantageous time for the correspondence. The *Journal of Comparative Neurology* directly assisted authors during different periods of the publication cycle and had excellent participation. The high rate of compliance is likely due to the direct assistance but also to the publication of an editorial to support awareness (Bandrowski et al., 2014) and a long‐standing history of antibody identification back to 2006. Neuroinformatics has section for tools, and several articles incorporated RRIDs even prior to staff looking them up. The *Journal of Comparative Neurology* also has such a section and antibodies.

Authors were willing to add resources to the registries if they were not available. We analyzed the statistics for the Antibody Registry and SciCrunch Tool Registries, as we had programmatic access to these. Since the project began, over 200,000 antibodies from vendors, both solicited and unsolicited, and at least 200 from individual authors were added to the Antibody Registry (antibodyregistry.org). In cases where antibodies are sold by government‐led projects such as NeuroMab from UC Davis, antibody identifiers have been included in the antibody manufacturer's Website. Many of the additions were secondary antibodies, which were not part of the pilot project but authors felt that they should also be identified. In one representative example, Jackson ImmunoResearch was contacted by several authors and subsequently submitted their full catalog to the Antibody Registry, allowing authors to report RRIDs for their secondary antibodies. Additionally, there were over 100 software tools and databases registered. Many were for common commercial statistical tools (e.g., SPSS, GraphPad), technically out of scope for the pilot project, but authors did not make the distinction between commercial and noncommercial tools. A comparison of new resources added versus those reported in the first 100 articles indicates that the Registries already listed the majority of research resources in each of these categories, as the number of new resources added for this set represented only ∼10% of the total reported resources.

Figure 2 shows the most common tools identified by RRID in articles from the first 100 articles. Commercial tools such as MatLab, SAS, and GraphPad were cited along with noncommercial tools such as ImageJ and FreeSurfer. The most common antibody was the anti‐NeuN antibody from Millipore, now Merck. These same resource identifiers have continued to be very highly cited in subsequent articles, with ImageJ cited in 42 articles and the NeuN antibody cited in 8 articles (Google Scholar March 17, 2015).

### Outcome #2: Performance

A major concern of the publishers and editors was whether or not authors could retrieve RRIDs correctly and whether significant editorial oversight would be necessary for quality control (see workshop outcome documents at https://www.force11.org/node/4857).

**Figure 3:**
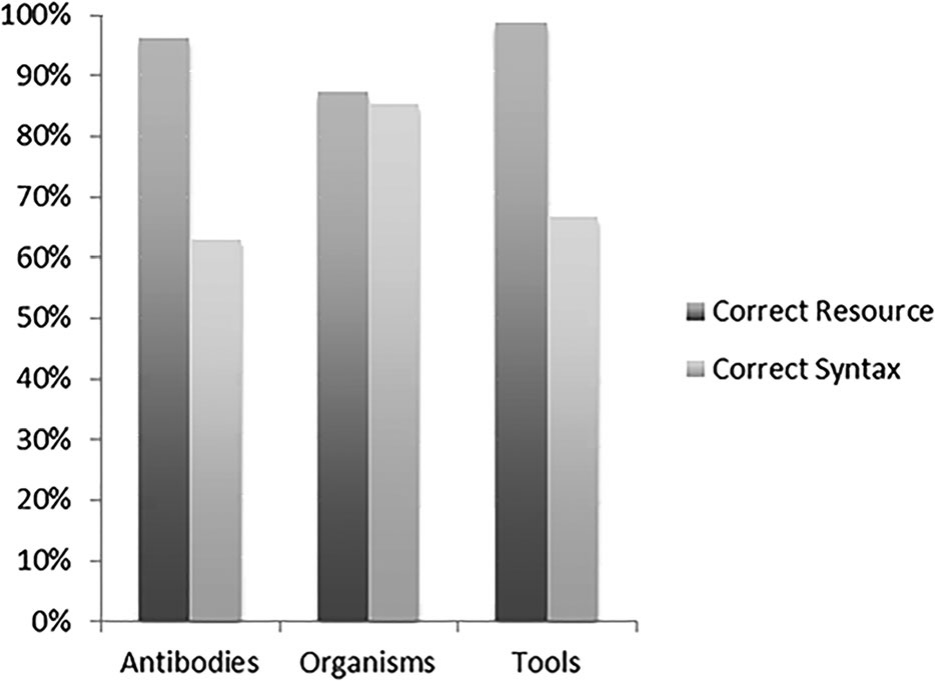
Percent correctly reported RRIDs. The percentage of resources that reported an RRID that pointed to the correct resource and with the correct syntax for each resource type is shown. The total number of resources for each type during the post‐pilot is: primary antibodies, *n* = 429; organisms, *n* = 55; noncommercial tools, *n* = 78.

To determine if authors were correctly reporting RRIDs, we analyzed the reported RRIDs and determined if they pointed to the correct resources in the RII portal, by comparing the metadata and RRID reported for each resource using the resolving service (for example, see https://scicrunch.org/resolver/RRID:AB_262044) or by querying the portal. Overall, 96% (538/562) of the RRIDs reported by authors were correct (i.e., the RRID pointed to the correct resource). More specifically, 96% of antibodies (413/429), 87% of organisms (48/55), and 99% of tools (77/78) were correctly reported (Fig. 3).

Inspection of the 16 errors in reporting RRIDs for antibodies (4% error rate) showed that three errors were copy/paste mistakes where authors mixed up the combination of catalog number and identifier for resources used in their article; three errors resulted from identifiers missing a digit at the end of the ID (for example, “Swant, catalog #6B3, RRID: AB_1000032” should have been labeled RRID: AB_10000320); and one error involved reporting a reference PMID instead of the resource identifier. The apparent cause of the other antibody errors was not possible to determine. For organisms, seven errors were made (13% error rate). All of these errors involved mice for which authors used the appropriate gene or allele identifier from Mouse Genome Informatics (MGI), rather than the stock number or genotype identifying the organism. It should be noted that MGI as of 2015 can search the genotypes, but at the time of the pilot project the search was limited to alleles, thus it stands to reason that authors went to MGI as opposed to the SciCrunch portal to identify resources. The fewest errors were made in identifying software tools and databases, with only one mistake from 78 total (1% error rate). The mistake was made as the author apparently used an antibody identifier instead of a tool identifier.

The use of a unique string to retrieve RRIDs is aided by a common syntax. In our analysis of RRIDs we also noted whether or not the RRID was correctly formed. In 66% (369/562) of cases the RRID was reported with the correct syntax, 63% of antibodies, 85% of organisms, and 67% of tools were formatted correctly (Fig. 3). The most common variant was the addition of extra spaces (RRID:AB_90755 vs. RRID: AB_90755), with 67% (129/193) of the minor corrections being due to an extra space. Other common variants were failure to include the RRID prefix, using various symbols or spaces in the identifier, or splitting up the RRID prefix and identifier in a table. Authors did not create RRIDs for resources they were either unable to find or were not in the portal in 142 cases, which constitutes an overall 20% false‐negative rate (36/465 reported antibodies were false negatives 8%, 84/139 reported organisms were false negatives 60%, and 22/101 tools 22% were false negatives). In other words, authors included RRIDs for the appropriate resource in over 80% of cases.

### Outcome #3: Identifiability

**Figure 4:**
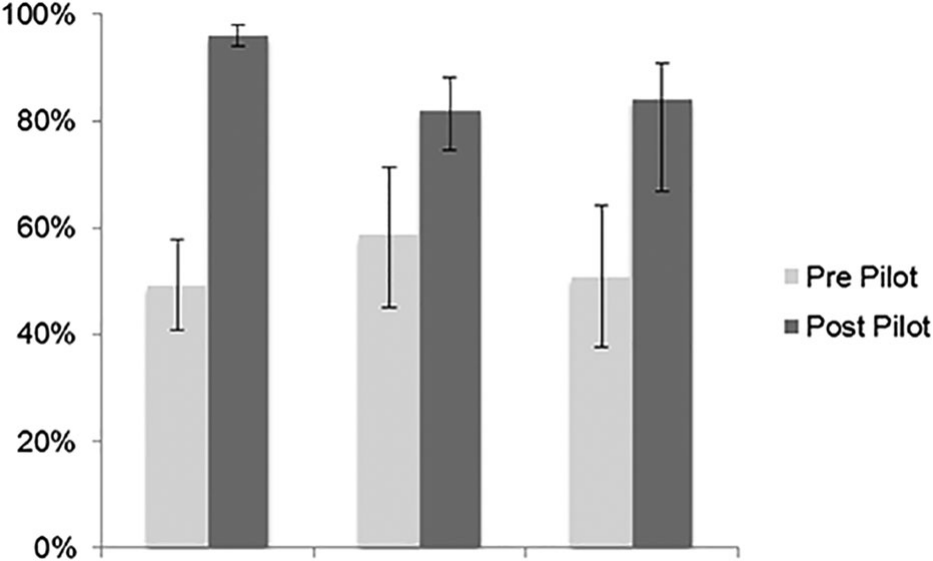
Pre‐ and post‐pilot identifiability. Resources (primary antibodies, organisms, and tools) were considered identifiable if they contained an accurate RRID or by using the same criteria as described in Vasilevsky et al. (2013). For tools (software and databases, which were not previously analyzed), these resources were considered identifiable if they contained an RRID or reported the manufacturer and version number. The total number of resources for each type is: primary antibodies pre‐pilot, *n* = 140; primary antibodies post‐pilot, *n* = 465; organisms pre‐pilot, *n* = 58; organisms post‐pilot, *n* = 139; noncommercial tools pre‐pilot, *n* = 59; noncommercial tools post‐pilot, *n* = 101. The y‐axis is the average percent identifiable for each resource type. Variation from this average is shown by the bars: error bars indicate upper and lower 95% confidence intervals. Asterisks indicate significant difference by a z‐score greater than 1.96.

An outcome of this study was to determine if the use of RRIDs in the literature increased the identifiability of research resources. As shown in Fig. 4, when authors were asked by their editors to provide RRIDs, regardless of their compliance with the RII project, the identifiability of research resources significantly increased. We calculated the percentage of identifiable research resources in the same journals, just before the pilot project and after. The reporting of research resources pre‐pilot was consistent with findings from the 2013 study (Vasilevsky et al., 2013), in that roughly 50–60% were found to be identifiable. But when asked by their editors, researchers used identifying information in 80–90% of research resources, showing that they presumably had the data available, but did not put it into their articles unless prompted by communication from the editors.

### Outcome #4: Utility

#### Machine‐processability

The ability to search all studies that used a particular research resource was a prime motivation for this pilot. The current project had a loose definition of “machine‐processable” because we did not want to impose any requirements on the publishers to modify their journal submission system for a pilot. Thus, we opted to craft RRIDs as unique, indexable alphanumeric strings based on authoritative sources that could support use of Web search engines to return articles using a particular research resource. We specifically asked authors to assess resources mentioned in the Materials and Methods section where they would normally provide identifying information, because we wanted to track actual use of the resource and not just mentions.

For individual RRIDs the approach was highly successful, as illustrated by the ability to type a particular RRID into three search engines for the biomedical literature: Google Scholar, PubMed, and Science Direct and retrieve appropriate articles, e.g., RRID:AB_90755 or AB_2298772 (for Google Scholar, see Fig. 2). It is important to note that each of these systems will come back with different results because each search tool has different types of data about each article. For example, ScienceDirect has a good full text search of all Elsevier content, but it does not search other publisher's content. Both PubMed and Scopus search only the abstracts and return a subset of articles where authors followed instructions to add RRIDs to the keywords, but not those that are only in the Methods section. Google Scholar is the most comprehensive, as it appears to search full text and brings back articles that are both published and unpublished (usually these are accepted for publication, but not yet indexed by PubMed). An analysis performed in October 2014 showed varying results from each search engine: Google Scholar returned 315 results (from 2014, 174 are true RRIDs), and ScienceDirect returned 18 (from 2014, three are RRIDs). PubMed revealed 23 articles that contained RRIDs (from 2014, all identify the resource identification initiative identifiers). Scopus returned 48 documents (from 2014, 18 are RRIDs).

**Figure 5:**
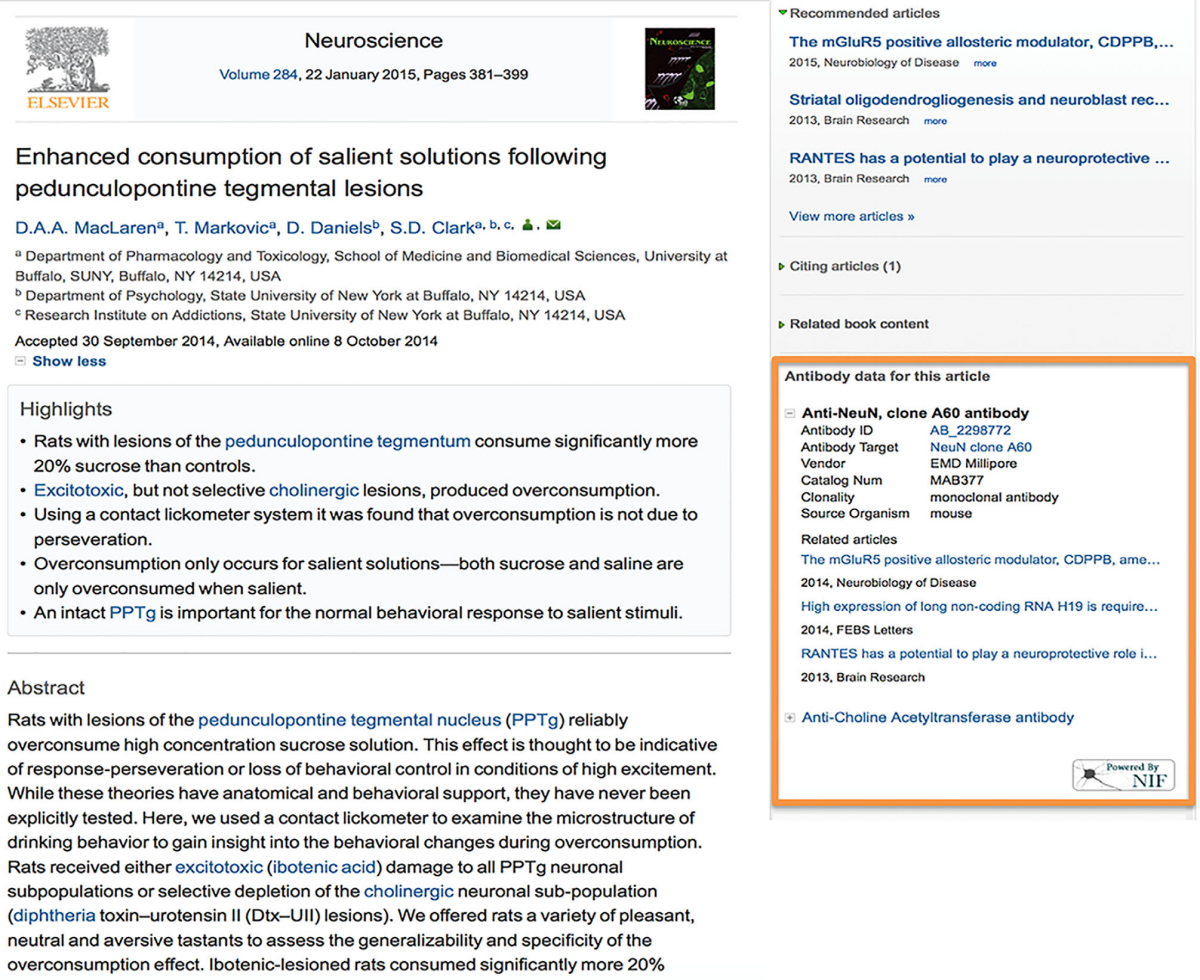
An exemplar third‐party application using the RRID resolving service. The “Antibody data for this article” application developed by Elsevier enhances articles on ScienceDirect. The application is available in 211 articles in 19 journals (more information can be found at: http://www.elsevier.com/about/content-innovation/antibodies).

To promote the development of third‐party tools around RRIDs, we created a resolver service for RRIDs using SciCrunch. Typing http://scicrunch.com/resolver/RRID:AB_90755 will resolve to a landing page with meta‐data on a particular entity. The resolving service allows applications to make use of RRIDs to, for example, enhance articles with RRIDs by providing additional information about the entity and link to relevant articles and resources. For instance, Elsevier has released their antibody application, which displays antibody metadata in the right‐hand side panel, next to the article (see Fig. 5 for a screen shot for MacLaren et al., 2015: http://www.sciencedirect.com/science/article/pii/S0306452214008458). The reader can browse through antibodies referred to in the article, view complete records in antibodyregistry.org, and access additional information via direct links to GenBank, ZFIN, and other relevant databases. The application also recommends the three most relevant articles published in Elsevier journals that refer to the same antibody. The application is freely available on ScienceDirect.

#### Publication practices

Non‐open‐access journals were asked to add RRIDs to publication keywords, but our initial findings suggest that this practice was not being consistently followed. Only 23 articles of 41 total (as of October 20, 2014) were accessible in PubMed. Additionally, it should be noted that in two cases identifiers were removed at typesetting after the initial online version of the article was published with the RRIDs. These identifiers were removed not only from the article, but also from PubMed keywords. Although this was reversed when noted by the working group, this demonstrates that successful implementation requires knowledge of the RRIDs and agreement by the publishers at all steps.

## DISCUSSION

The pilot RRID project has been highly successful in demonstrating the utility of a system to aid in the identification of antibodies, software and databases, and model organisms in the biomedical literature. We showed that authors were willing to adopt new styles of citation for research resources that promoted more accurate identification of research resources used in a study, and that were more amenable to machine‐based identification. To date, RRIDs have appeared in over 400 articles from 60 journals. With one exception (the *Journal of Neuroscience*), journals have continued their request for RRIDs beyond the initial 3‐month pilot project and new journals have signed up beyond the initial set that started the project. We believe that the success of the project was due to the extensive preplanning that involved the publishers and the editors, the limited scope of the initial request, and the recognized need by researchers for better and more useful reporting standards for research resources.

The load on curation staff with participating journals has been minimal and the initial portal prototype appears reasonable for the majority of authors to find their resource identifiers. With >10,000 searches in the RII portal, there were ∼100 help questions. Many of these questions were about scope, i.e., whether a particular research resource should be identified, others were for assistance in finding a resource or guidance in adding a resource not yet contained in the community authorities. While this is not a large number, it is also not insignificant, particularly as the project expands, and certainly points out the need for specific help functions.

Given the relative completeness of the registries and the rapid advance of machine‐learning based techniques for entity recognition, we can envision a semiautomated system that assists the author in supplying correct IDs. We have already improved our ability to detect digital research resources in the literature using machine learning (Ozyurt et al., in review, *PLoS One*). In this system, machine learning is used to identify software tools and databases in text and compare the information to Registry listings. The development of such functions would allow the development of recommender systems for authors and automated fact checkers for journal staff.

### Why unique identifiers?

Unique identifiers serve as a primary key for identifying a given research resource and providing the ability for search engines to parse them is paramount. Unique identifiers enable disambiguation of entities with similar labels. The ID should not point to two different entities and needs to be persistent, that is, they need to outlive the entity itself. They also need to be at least minimally machine‐processable. While many authors supplied identifying information like the catalog number for an antibody supplied by the vendor, or the official strain nomenclature supplied by the IMSR for a mouse, neither of these served the required functions. A catalog number is not a unique identifier, but rather a useful way for vendors to identify their products. If different vendors sell the same antibody, it will have different catalog numbers. If the same antibody is sold in different aliquots, it may have different catalog numbers. When the antibody is no longer available, the catalog number may disappear, or in some cases it may be reassigned to another antibody. All of these features are undesirable in an identifier system. The Antibody Registry, in contrast, was specifically designed to supply useful and stable identifiers for antibodies and not as a commercial source of antibodies. Similarly, the strain nomenclature developed by the Jackson Laboratory, with its superscripts and special characters, is useful for human curators to identify a particular strain, but causes hiccups in most search engines because of all of the special characters. We believe that a well‐curated registry is essential to the success of such a system, because of the necessity of these two functions, which currently cannot be replaced with a simple uncurated registration service. For example, we found in the registries we maintain, both software or antibodies, that authors sometimes register an entity that is found by a curator to be a duplicate.

### Reporting of RRIDs

When considering accuracy and syntax, the majority of the issues were due to minor syntax errors (33% of RRIDs had a syntax error), and a minority of the resources (4%) was incorrectly reported. The data suggest authors are able to find the correct RRID for their resource, but the higher syntax error rate indicates a need for an improved process for reporting the RRIDs in the manuscripts. Typesetting may cause some of the syntax issues, for example, spaces may be introduced, especially when the RRID is at the end of a line. Additionally, these types of syntax errors are resolvable by the resolver, so they do not pose an issue for the machine readability.

Authors included RRIDs for the appropriate resources in 80% of the articles. This analysis did not allow us to determine if authors did not report RRIDs because the resource was not available in the RII portal at the time, or if they failed to include the RRID for another reason.

The analysis for this pilot project focused on primary antibodies and noncommercial tools; however, many authors included RRIDs for secondary antibodies and commercial tools, such as MatLab or SAS. While this was out of the scope for this analysis, this indicates that authors are willing and eager to provide RRIDs for additional research types, not just those included in this pilot project.

In two articles, authors reported RRIDs for resources that were not used as part of the study, but rather were discussed in the Introduction or Discussion sections. A goal of this study is to enable one to determine the usage of a particular resource, as reported in the published literature. For example, one could query Google Scholar for all the articles that report a particular RRID to get a sense of how frequently that resource appears in publications. Therefore, it is important that only resources that are used in a study are assigned an RRID. This should be further clarified in the instructions to authors.

### Which identifiers?

There are many types of and formats of identifiers in use today (e.g., DOIs, URIs, ARCs), each with varying amounts of associated infrastructure and use in different communities. For this project, we elected to use simple alphanumeric strings and a common syntax in the form of accession numbers issued by the *authoritative* community‐based registries. We relied on each registry to impose the uniqueness constraint at the level of the entity, for example, ensuring that there was only one mouse genotype per unique ID, and to ensure standard metadata by curating each entry. The reuse of authoritative accessions with the RRID prefix provides maximal flexibility and interoperability and minimal ID churn, while also provisioning for resource identification.

A frequent question regarding the RRID is why we did not use a DOI as a unique identifier instead of the Registry Accession number. Part of the reason was cultural: researchers were used to supplying accession numbers for GenBank, Gene Expression Omnibus, Protein Data Bank, etc., and understand this requirement. Part of the reason is practical: unlike DOIs, accession numbers are already available for most of the research resources to be identified in this pilot and did not require special infrastructure to resolve or cost to issue. Part of the reason is also philosophical: DOIs are for digital objects, such as individual articles, that live on the Web and need to be resolvable. A DOI resolves to a particular article that is self‐contained—it is the object. In contrast, an antibody does not exist on the Web but is an independent entity that has data about it scattered across various articles. There is no single digital record that is the antibody; there are documents and data about the entity. We note that in our community we also do not use DOIs to identify people, but rather an ORCID, which serves the same purpose as the RRID.

A case could be made for using DOIs to identify particular software tools and databases, as they are digital objects. As discussed in the next section, our preference is that DOIs be used to identify the particular instance used, e.g., the version of data or software and any supporting workflows, and that the RRID be used to identify the entity or project referenced. Thus, the RRID would be used to identify the Protein Databank, and a PDB identifier or a DOI used to reference the specific data from the PDB. However, we believe that as the RRID system is adopted, each community should set appropriate identifier systems. The RRID syntax is meant to be simple and generic and could, in theory, work with any existing authoritative identifier system.

### How granular should RRIDs be?

RRIDs are meant to identify research resources at a fairly high level of granularity. At some of the planning meetings, there was a push for more granular information, e.g., lot and/or batch numbers for antibodies. We recognize that this level of granularity is likely an important factor in determining how a given reagent performs (Slotta et al., 2014). In our analysis by Vasilevsky et al. (2013) and in our experience using text‐mining, the biggest problem is not that authors were not supplying lot numbers but that they are not even supplying the minimal identifying information such as catalog numbers. Given that the catalog numbers themselves do not serve as stable identifiers, because antibodies are bought and sold and redistributed by many vendors, we elected to tackle the problem of identifying the root antibody first, i.e., a particular clone for a monoclonal antibody or a type of polyclonal antibody produced by particular protocol. To illustrate the problem, consider the study by Slotta et al. (2014) that provided an analysis of the performance of antibodies to NF‐κB‐subunit p65, as a follow‐up to a similar study by Herkenham et al. (2011). Both studies performed specificity tests on a variety of antibodies and, as is common, did not produce concordant results on all of them. Slotta et al. originally generated the antibody now commonly known as MAB3026 (AB_2178887) and provided its provenance: “It was transferred to Boehringer‐Mannheim as Clone 12H11, resold to Roche and finally bought by Chemicon, and it is now sold as MAB3026.” They then speculate that a mutation may have crept in at some point that altered the specificity of the antibody. However, the discrepancies may also be attributed to the additional testing of the antibody in new conditions, revealing problems that had not been apparent during the initial applications. The authoritative Antibody Registry identifier (and therefore the RRID) for this antibody combines these different representations together so that all references to this antibody can be tracked. Authors are encouraged in the citation format to include details about the particular instance of this antibody, namely, the vendor from which the antibody was purchased and the catalog, batch, and lot numbers. However, we did not want to overload the ID system to require assignment of these different lot numbers different RRIDs and maintain the mappings. We were also concerned that this would grossly decrease compliance.

For organisms, all of the authors' “errors” were due to the allele being reported but not the organism stock or genotype. The allele ID is not sufficient for identifying the animal used, as the same allele may be inserted into different mice of various backgrounds and with other alleles, and therefore will have different phenotypic characteristics. It should also be noted that authors consulting the MGI database (up to October 2014), which maintains the authoritative mouse nomenclature, would be able to search for MGI identifiers for genes and alleles, but not genotypes. This shows that authors likely went to MGI to obtain their identifiers rather than searching the RII portal, but were not able to find the genotype information and substituted the allele ID. MGI now searches the genotype information for all mice, suggesting that authors of newer articles can now also find the genotype information more easily at MGI and a tutorial for how to obtain a genotype identifier from MGI is now posted on the RII portal pages. Support for genotype identification, and therefore RRIDs, is planned to also be provided by a new Monarch Initiative phenotyping tool for submission of genotype–phenotype data to journals and model organism databases.

For tools (software and databases), we elected to identify the root entity and not a granular citation of a particular software version or database. Our main goal in the case of software tools and databases was to track broad patterns of utilization of these resources (e.g., how many times NeuroMorpho.org was used) and not particular versions. More complete practices for citing software and datasets are emerging from recent efforts like the Joint Declaration of Data Citation principles (https://www.force11.org/datacitation), the W3C HCLS dataset description (http://tiny.cc/hcls-datadesc), the software discovery index (http://softwarediscoveryindex.org/), and many others. These groups are exploring more complete reporting standards for the individual instances (versions, workflows, virtual machines) that can be used to reproduce the findings. We note that the goal of using RRIDs for software tools was to determine participation rates for authors identifying these resources using the easiest possible solution, with the longer‐term goal including more robust versioning and archival software practices that would support reproducibility.

### What are the next steps?

The RII is a grassroots effort that took advantage of existing investments by the NIH to solve a problem without extensive new infrastructure. The RII is continuing to run and has expanded beyond the initial participants. We believe that the growth of the initiative indicates that it fills a need not currently met by our existing practices and infrastructure.

Should RRIDs be adopted broadly across all of biomedicine? We would argue yes, the RRID syntax should become the standard for reporting on usage of research resources. We have shown that the requirements for this type of broad adoption are the availability of comprehensive and authoritative registries for the appropriate entities, a centralized portal or services that aggregate these registries into a single search, and the willingness of a community including journals and publishers to support this type of reporting. More sophisticated services can be built to improve and automate authoring and editorial oversight, but these are not required. The solution is therefore accessible both to large commercial publishers and smaller community‐ or society‐based journals.

If RRIDs were to be broadly adopted tomorrow, what are the outstanding issues regarding implementation and scalability? The first issue is one of scope. The current RII focused on three types of research resources that were broadly used and a known source of variability within experiments. Should all research resources be similarly identified, i.e., every chemical, instrument, etc.? We think such an approach would be clumsy and difficult to implement. We can imagine a future where all reagents and tools are barcoded and scanned as they are used in a study. However, as long as humans are responsible for supplying identifiers, we think that the effort should focus on certain types of known problematic entities for which better metadata and ability to query across articles is required. Given the recent problems associated with certain cell lines, for example, these are obvious candidates (ICLAC, 2015). The advantage of the current system is that it allows communities who have taken the steps to aggregate and organize resources that are of use to them to agree to include the RRID syntax and single entry point.

The second issue is governance. We deliberately designed a decentralized system that gives control of issuing identifiers to multiple authorities. Such a model requires some governance, in the form of willingness of the authorities to maintain the integrity of any identifiers and links and implementation of a policy regarding entities that are no longer available. We would also need some governance to ensure that multiple, uncoordinated authorities are not issuing IDs for the same research resource and that the IDs assigned to each entity are unique. The latter constraint is handled by the centralized aggregation service currently provided by SciCrunch; however, it may be handled by other services in the future. Further, the RRID project promotes consistent citation of research resources at a first level identifiability. We believe that more granular reporting standards can and should work hand in hand with the RRIDs and could be coordinated with the authoritative communities; for example, versioned software releases in GitHub.

Some of these governance issues are necessarily interdependent on issues relating to sustainability. As we increase participation among journals and resource providers, it would make sense to spread the cost of maintenance and development. One thing to consider is that resolution services can provide advertising for resource providers as third‐party applications are developed to connect people to resources in different contexts (such as in the Elsevier application described above). We would conjecture that as the number and types of these applications increase, the need to contribute and therefore help sustain resource registries will become increasingly advantageous.

We believe that the RRID project lays an important foundation for creating a type of “universal product code” (UPC) to help alert the scientific community when issues are raised about key research resources. Reagents and tools are not perfect and problems can arise, as the resources themselves can have issues as they are tested across various paradigms and systems. Even when a resource initially performed well, due to spontaneous mutations in biological resources and interactions between particular software tools and platforms, problems can arise over time. For example, two recent articles have published extensive tests showing that common antibodies for NF‐κB show nonspecificity under some circumstances (Listwak et al., 2013; Slotta et al., 2014). Many of these antibodies are extensively used in the literature, but readers of a particular article have no way of knowing that concerns have been raised. We have similar examples with software tools (Gronenschild et al., 2012), datasets (Hupé et al., 2015) (Button et al., 2013), and genetically modified animals (Cone et al., 2013). We have an infrastructure in place, CrossMark, to alert readers of a particular article that an addendum or erratum has been posted. The RRID system can serve as the basis for a similar system for research resources.

## Supporting information

Supplementary Information
